# The Medical Chronicle, or Montreal Monthly Journal of Medicine and Surgery

**Published:** 1853-07

**Authors:** 


*The Medical Chronicle, or Montreal Monthly Journal of Medicine and Surgery.* Edited by WM. WRIGHT, M.D., & D. G. MACCALLUM, M.D., Montreal, Canada.

THE Canada Medical Journal is but just dead, and behold, the Chronicle as if arising from the very ashes of its predecessor. Verily, if medical journals were monsters, we might conclude that in Montreal there is a hydra; no sooner is one head dead than another sprouts up. We hope this will not die from starvation. It deserves a better fate. Price $2 per annum, payable in advance. J.

